# Exploring the connection between maternal mental health and partnership, parental role, and satisfaction with various aspects of life using pairfam data: a cross-sectional analysis

**DOI:** 10.1186/s12905-025-03933-7

**Published:** 2025-08-18

**Authors:** Monique Förster, Claudia Kirsch, Julia Habermann, Dorothee Noeres

**Affiliations:** Hannover Medical School, Medical Sociology Unit, Carl-Neuberg-Straße 1, Hannover, 30625 Germany

**Keywords:** Mental health, Maternal mental health, Mental health composite scale of SF-12, Pairfam, Partnership, Parental role

## Abstract

**Background:**

Decreased maternal mental health leads to difficulties for the mother herself and for her family life. In Germany, inpatient mother-/father-child preventive and rehabilitation clinics are addressing these parental health problems. Further analysis, however, is needed in order to better understand the origins of impaired parental health and to improve the interventions. The present study focuses on maternal mental health and its association with strains related to mothers’ parental role, their partnership, and satisfaction with various aspects of life.

**Methods:**

For this cross-sectional study data from the relationship and family panel *pairfam*, wave 11, were used. Mothers in a cohabiting relationship with at least one child living in their household were examined. T-tests were employed to compare mentally stressed and mentally not stressed mothers in terms of (1) partnership (disagreements with partner, own destructive conflict behaviour, partner support and recognition), (2) parental competence, (unspecific strain, missing autonomy, and overprotectiveness in the parental role), and (3) satisfaction with work-life balance, leisure activities, friends and social contacts, and family. Multiple linear regression analysis was conducted, with the mental health composite scale of the Short Form 12 (Version 2.0) Health Survey as the dependent variable, and the previously mentioned variables as independent variables.

**Results:**

Among 1,441 mothers in partnership, 153 (10.6%) were mentally stressed. Mean comparisons indicated significantly poorer values for mentally stressed mothers across all variables. The results of the linear regression model demonstrated a correlation between maternal mental health and disagreements with the partner (B=-1.318, *p* =.002), own destructive conflict behaviour (B=-1.232, *p* =.002), parental competence (B = 1.606, *p* <.001), unspecific strain (B=-1.402, *p* <.001), missing autonomy (B=-0.732, *p* =.030), overprotectiveness (B=-1.015, *p* <.001), and satisfaction with work-life balance (B=-2.537, *p* =.003), and family (B = 0.432, *p* =.029).

**Conclusions:**

The findings of this study are consistent with the existing literature, indicating that parental role has the strongest connection with maternal stress. Additionally, novel findings have been identified, including the significant associations of partnership conflict and satisfaction with work-life balance and maternal mental health.

**Supplementary Information:**

The online version contains supplementary material available at 10.1186/s12905-025-03933-7.

## Background

Mothers may experience various burdens in their parenting and family life, including permanent time pressure, financial burdens, mental and physical stress, and burdens of partnership [1]. In addition, there is a possible mental spillover effect between work and family, meaning that they think about job-related matters while doing care work and vice versa [2, 3]. Mental disorders have a significant impact on the individual and their environment. The most recent 12-month prevalence of mental disorders in Germany was 27.7%, in 2011, with women demonstrating a higher prevalence compared to men (33.5% vs. 22.1%) [4, 5]. Maternal mental health has been demonstrated to exert a significant influence on the lives of both the mother and her child(ren), as well as the dynamics of the partnership. The consequences of maternal mental illness for the mother herself can be considerable, including a loss of healthy life years, a reduction in life expectancy, and the necessity for early retirement [6–8]. Concomitantly, children of mothers with poor mental health are predisposed to manifesting diminished social-emotional, cognitive, language, motor, and adaptive behaviour development [9, 10]. Moreover, evidence suggests that children demonstrate an elevated prevalence of health service utilisation and poorer physical health outcomes, including infectious diseases, pathological abdominal disorders, and atopic dermatitis [11]. However, positive maternal mental health has been demonstrated to weaken the negative association between socioeconomic status and the child’s mental health, as well as the association between socioeconomic status and the child’s cognitive abilities [12]. Furthermore, a positive relationship has been observed between maternal mental health and children’s mental health and social functioning, thereby indicating a connection between maternal mental health and child well-being [13]. The correlation between maternal mental health and partnership is primarily investigated during the antenatal period. In this context, a correlation has been demonstrated between partner support and prenatal depression and anxiety, thus indicating the significance of the partner’s role [14].

Moreover, mental disorders incur considerable expenses for healthcare systems. In Germany, in 2023, mental illnesses were the most prevalent cause of early retirement, accounting for 42% of cases [8]. Furthermore, 16% of sickness absences were associated with mental illness, resulting in production losses amounting to 20.5 billion euros [15].

As demonstrated in the literature, elevated levels of depression and a negative association between parental self-efficacy and maternal depression have been observed to be prevalent among parents with a lower level of education and a lower household income [16, 17]. Furthermore, there is a growing body of literature relating to women’s health risks, which are attributed to the physical, socio-emotional and cognitive tasks that women carry a dis-proportionately high share [18, 19]. These factors may be partly related to an effort-reward imbalance in household and family work [20], or simply to the multiplicity of physical, mental and cognitive labour at home [21].

In Germany, inpatient mother-/father-child preventive and rehabilitation clinics are specialised in supporting parents with (potential) health problems, with a particular focus on the parent-child relationship and addressing the needs of parents experiencing health challenges. Every mother or father is legally entitled to apply for a mother- or father-child measure, which is mostly funded by a medical insurance and has to be prescribed by a medical doctor. The implementation of this measure is subject to the presence of certain context factors [22], which can be categorised as either general or maternal/paternal [23]. The former include time pressure, financial difficulties, restricted housing conditions, and social isolation, while the latter encompass parenting difficulties, single parenthood, children with disabilities or behavioural problems, and an impaired parent-child relationship [23]. The three-week intervention programme incorporates childcare, counselling and various therapies addressing physical and mental health issues, parent-child interaction, lifestyle, and parenting behaviour. Research indicates that up to 80% of mothers in inpatient mother-child measures show clinically relevant psychological stress [24]. Consequently, 54.2% of the mothers participating in such a measure demonstrate elevated stress levels, 26.2% exhibit heighened fear, and 23.0% encounter augmented strain due to depression [25].

Whilst the conceptual clarity of the inseparability of maternal emotional well-being from other areas of life, such as family and work, is evident, further analysis is required in order to ascertain the attitudes and effects of social relationships on maternal mental health. This, in turn, will provide the necessary information for the development of evidence-based solutions that will improve the quality of life of mothers.

The present study firstly aims to better understand the circumstances of maternal strain by examining the differences between mothers experiencing mental distress and those not experiencing such distress focusing on the following three areas: (1) partnership, (2) parental role, and (3) satisfaction with various aspects of life. Secondly, in order to identify possible areas of intervention, this study aims to identify relevant factors that might be associated with maternal mental health, focusing on the three areas of mothers’ lives mentioned above. Inpatient mother-/father-child prevention and rehabilitation clinics are intended to serve a number of purposes, including the provision of support for mothers experiencing mental health challenges who find themselves confronted by the topics addressed in this study. The research adopts an interdisciplinary perspective that integrates socio-structural, psychological, and sociocultural dimensions of motherhood.

## Methods

### Study design

In this cross-sectional study, a secondary data analysis was performed using data from the German ‘Panel Analysis of Intimate Relationships and Family Dynamics’ (*pairfam*), release 13.0 [26]. This panel constitutes a multi-disciplinary, longitudinal study with a multi-actor design, initiated in 2008/2009 with anchors drawn from the birth cohorts 1971–1973, 1981–1983, and 1991–1993. From wave 11 onwards, an additional birth cohort 2001–2003 was included. In addition to the anchor persons, the respective partners have been interviewed separately from the first wave onwards, the (step-)parents from the second to the eighth wave, and children of the anchor persons from the second wave onwards. The initial sample with 12,402 anchors and 3,743 partners was obtained through two stages of data collection. Firstly, municipalities were drawn within the Federal Republic of Germany. Secondly, it was drawn from the population of individuals who are registered as having their main place of residence in the selected municipalities. The objective of *pairfam* is to provide an empirical foundation for enhanced comprehension of the dynamics prevalent within couples and families. The anchors are interviewed using a Computer-Assisted Personal Interview (CAPI), which includes a Computer-Assisted Self-Administered Interview (CASI) for sensitive questions. A Paper and Pencil-Questionnaire (PAPI) is utilised to survey the partners, while a 15-minute CAPI is employed for the children. The (step)parents were surveyed by mail [27]. For a comprehensive overview of the conceptual framework, methodology, sample sizes and further details of the survey, see Huinink et al. [27] and Brüderl et al. [26].

### Participants

The sample for the ensuing analysis was derived from wave 11 (2018/2019, *N* = 9,435), with two rationales underpinning this selection. Primarily, all the selected variables were surveyed in this wave. Moreover, at the inception of the study, this wave was the most recent prior to the onset of the coronavirus pandemic, thereby ensuring that the participants’ responses were not influenced by the circumstances of the pandemic.

The inclusion criteria for the analysis encompassed mothers with a cohabiting partner and at least one child under 18 years in the household.

**Fig. 1:**
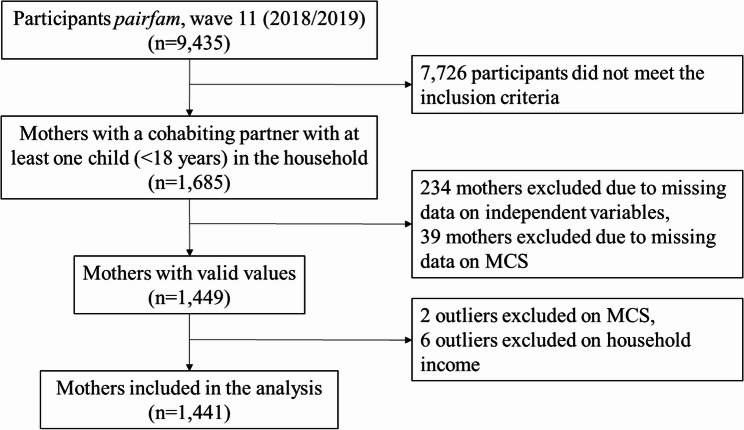
Flow chart of the participants in the study

Mothers living apart together, defined as mothers in a relationship in separated households, and single mothers were excluded from the study due to their particular experience of stress related to their family situation. Single mothers are likely to experience elevated levels of stress [28], due to their circumstances, they cannot resort to a potentially supportive partner or share duties and responsibilities. Additionally, mothers with missing data were excluded, and outliers were identified and subsequently excluded using z-standardisation with values below − 3.29 and above 3.29 [29] (Fig. 1). The resulting sample included 1,441 mothers.

### Study variables

#### Maternal mental health

The dependent variable, i.e. maternal mental health, was measured by the mental health composite scale (MCS) of the Short-Form 12 (Version 2.0) Health Survey (SF-12). The SF-12 is a short version of the SF-36, aiming to determine the health-related quality of life. It has been demonstrated to be valid for the German population [30]. The instrument comprises 12 questions, which are allocated to eight subscales and two dimensions of physical (PCS) and mental health (MCS). (For a detailed description of the instrument, see additional file I.) The four subscales belonging to the physical health dimension include “physical functioning”, “role physical”, “bodily pain”, and “general health”, the other four subscales represent the mental health dimension, i.e. “vitality”, “social functioning”, “role emotional”, and “mental health”. Despite the allocations to two different dimensions, all eight subscales were used for the generation of both dimensions, meaning that the four subscales for physical health were also used for the generation of the mental health indices MCS (however, with a lesser weight) and vice versa [31]. The items are measured on three-step to five-step scales, ranging from “strong” to “not at all” for the three-step scale, from “hardly ever” to “almost always” for the four-step scale, and from “bad” to “very good” for the five-step scale. The calculated MCS is included in the *pairfam* dataset [32]. The MCS is calculated by determining the mean values of the eight subscales. Subsequently, each subscale is standardised using the z-transformation to a scale ranging from 0 to 100. In the subsequent step of the process, these scales are combined with a weighting to form the MCS [31]. The MCS is also transformed into a norm-based score with values ranging from 0 to 100 (mean = 50, SD = 10). Consequently, a high value on the scale is indicative of a high health-related quality of life or, respectively, of a low health-related restriction.

The classification of mothers as either mentally stressed or mentally not stressed was determined by the mean values of their MCS scores, with a cut-off value established by subtracting one standard deviation (10.0) from the mean value (M = 50), as recommended in the manual of the SF-36 [33], relating to the respective overall norm sample of 1998. These values (Mean and standard deviation) are similar to those determined on the basis of a norm sample of the German population in 2004, with women aged from 18 up to maximum age showing slightly lower values (M = 48.94, SD = 10.21) than men (M = 51.14, SD = 9.63) [31], but using a slightly altered version of the original instrument. The SF-36 and the SF-12 have been shown to be comparable in their results [33]. Consequently, we followed the original recommendation [33] and designated mothers with a value less than 40 on the MCS as mentally stressed, while those with a value greater than or equal to 40 were categorised as mentally not stressed.

#### Partnership

In order to assess the situation in the partnership, the variables “disagreements with partner”, “own destructive conflict behaviour”, and “partner support and recognition” were examined. For the variable “disagreements with partner”, questions were asked on the frequency of disagreements in six different areas (for further detail on all variables see the additional file I). The variable “own destructive conflict behaviour” was measured by the query, how often the anchor engages in six different behaviours in case of disagreement. The variable “partner support and recognition” asked how the anchor felt as a parent, with two corresponding items: one covering partner support and one appreciation. Mean values from the respective items described here, as well as in the additional file, were used.

#### Parental role

In determining the parental role, the variables of “parental competence”, “unspecific strain”, “missing autonomy in the parenting role”, and “overprotectiveness” were examined, each comprising multiple items with scales also ranging from 1 “not at all” to 5 “absolutely”. The variable “parental competence” encompasses four statements measuring the anchor’s perception of the role as a parent. The variable “unspecific strain” represents the extent to which the anchor agreed with the statement “My life with child(ren) is exhausting” and “I am often at the end of my rope”. The variables “missing autonomy” and “overprotectiveness” were measured using four and three items, respectively, based on the extent to which the anchor agreed with the respective statements. Again, the means of the items belonging to the respective variable were used (for further detail on all variables see the additional file I).

#### Satisfaction

Three areas of respondents’ satisfaction were measured: “leisure activities, hobbies, interests”, “friends, acquaintances, social contacts”, and “family”. A fourth item examined their satisfaction with their work-life balance, asking “how satisfied are you with the proportion of time that you spend on the job or for your vocational training or university education relative to the time that you spend on your personal life?” (see additional file I). For the latter item, a missing data dummy variable was created for the regression model, in order to avoid the exclusion of unemployed women or students or trainees. These women constitute one of three comparison groups (see Table 3).

### Covariates

As there is sufficient evidence for the correlation between the socio-economic status and mental health [34–36], education, average weekly working hours, migration background and household net income are used as control variables. Equally various lifestyle factors such as smoking, alcohol use and physical activity have been shown to be related to mental health [37] and were also used as controls. Education is analysed according to the classification scheme originally established by the Project on Comparative Analysis of Social Mobility in Industrial Nations (CASMIN). In *pairfam*, the updated version of the original classification was applied [38]. Both school-based and vocational degrees are considered. The calculated values according to CASMIN are included in the *pairfam* dataset. For the current study, the classification scheme was summarised as low, middle, and high education.

The role of paid work is determined by the number of average weekly working hours surveyed by the following question: “What, on average, are your actual weekly working hours, including overtime? For this calculation, please take into account all of your jobs.” The resulting number is documented in hours per week. Women who are not engaged in paid employment were assigned a zero.

Migration background reports whether the anchor has a migration status and, if applicable, to which generation of migrants the anchor belongs. The categories in the pairfam dataset include: “no migration status”, “1st generation”, and “2nd generation”. We combined 1 st and 2nd generation to 1 “no migration background” vs. 2 “migration background” in the respective variable.

The evaluation of household income is based on the net equivalence income according to the modified equivalence scale of the Organisation for Economic Co-operation and Development (OECD). It is calculated by dividing the monthly household’s net income by the equivalence scale weight. The latter is determined on the basis of the age structure in the household, with the first household member counting as 1, other adults counting as 0.5, and children under 14 counting as 0.3. In instances where the household net income information was unavailable, no variable values could be generated. The determined values of the net equivalence income according to the modified OECD equivalence scale are included in the *pairfam* dataset.

Variables controlling for lifestyle factors included whether the respondent is actually smoking, the frequency of alcohol use, and the frequency of physical activity. All variables utilised in this study are drawn from the *pairfam* data set [26, 39].

### Statistical analyses

Firstly, descriptive analyses were performed. Secondly, the two groups of mothers– mentally stressed and mentally not stressed– were compared by calculating t-tests for independent samples. The test variables included ratings of (1) the relationship with the partner, (2) the parental role, and (3) the satisfaction with various aspects of life. Thirdly, the same variables together with the control variables education, average weekly working hours, migration background and household net income were included as independent variables in a multiple linear regression model with maternal mental health as dependent variable. A second model, including lifestyle and socioeconomic factors as covariates was calculated, but not displayed here. Testing the necessary assumptions linearity, independent errors, homoscedasticity, normally distributed errors, multicollinearity [29], we found no assumptions violated, still we conducted bootstrapping (1,000 samples) with replacement for more robust confidence intervals (95%) and excluded user-missing values.

A power analysis was conducted to ascertain whether the sample size was adequate for detecting medium-sized effects [39]. For the group comparisons via t-tests (n1 = 153, n2 = 1,288), the achieved power was > 0.99 for detecting medium-sized effects (d = 0.5), exceeding the conventional threshold of 0.80. Similarly, for the multiple regression analyses with 21 independent variables, the power to detect medium-sized effects (f²=0.15) was > 0.99, again affirming the adequacy of the sample size (*N* = 1.441) for the conducted analyses. Following the rule of thumb that 10 cases per independent variable are needed for adequate power to detect significant individual independent variables in multiple linear regression, the current sample size (*N* = 1,441) far exceeded the minimum requirement of 210 cases (21 independent variables ×10), further supporting the high statistical power of the analyses.

All statistical analyses were performed with SPSS 29.

## Results

### Sample

The present study’s sample consists of 1.441 mothers in cohabiting partnerships aged between 24 and 48 years (M = 37.6; SD = 6.1). The mean number of children is 1.9 (SD = 0.8), with 15.5% of mothers having three or more children living in their household. For 43% and 35% of mothers, respectively, the youngest cohabiting child is toddler or school age. More than half of the participating women (53.6%) have attained a medium level of education, and the average weekly working hours are 20.1 (SD = 16.0). However, more than one in four women do not work at all, while slightly less than one in four works more than 35 h per week on average. The household net equivalence income according to the modified OECD equivalence scale ranges from €104.17 to €5,000.00 (M = 1,851.13; SD = 723.44), with almost one quarter of the sample having an income below €1,500 (Table 1).

The mental health scores of the respondents vary from 8.4 to 76.0 on the MCS scale, with a mean of 50.2 (SD = 9.0). While 153 mothers (10.6%) were categorised as mentally stressed, 1,288 (89.4%) were considered as mentally not stressed (Table 1).

**Table 1:** Sample description: mothers in cohabiting partnership with at least one child under 18 in household

	***N***	**%**	**M (SD)**
Age			
24-28	181	12.6	37.6 (6.1)
34-38	866	60.1	
44-48	394	27.3	
Number of all children living with anchor			
1	457	31.7	1.9 (.8)
2	707	49.1	
3	223	15.5	
4 up to 8	54	3.7	
Age of youngest cohabiting child			
infant (0 months to under 1 year)	169	11.7	6.0 (5.0)
toddler (1 to under 6 years)	617	42.9	
school child (6 to under 14 years)	502	34.9	
teenager (14 up to and including 17 years)	153	10.6	
Education			
low	158	11.0	
middle	772	53.6	
high	511	35.5	
Average weekly working hours			
none	414	28.7	20.1 (16.0)
low (up to and including 10 hours)	77	5.3	
middle (11 to less than 35 hours)	615	42.7	
high (35 to 80 hours)	335	23.2	
Household net equivalence income (according to OECD)			
104.17 to under 1,000 Euro	114	7.9	1,851.13 (723.44)
1,000 to under 1,500 Euro	339	23.5	
1,500 to under 2,000 Euro	472	32.8	
2,000 to under 2,500 Euro	297	19.9	
2,500 up to 5,000 Euro	229	15.9	
Migration background			
migration background	331	23.0	
no migration background	1110	77.0	
Mental health composite scale (MCS)			
mentally not stressed (40 up to and including 76.0)	1.288	89.4	50.2 (9.0)
mentally stressed (8.4 to under 40)	153	10.6	
	1.441	100.0	

### Mean comparisons

Mentally stressed mothers show a significantly lower level of functionality in their partnership and parental role, as well as a decreased satisfaction with various aspects of life, in comparison to their mentally not stressed counterparts (*p* <.001, see Table 2). The most pronounced disparity concerning partnership characteristics between mothers experiencing mental distress and those not experiencing it is observed in “disagreements with partner” and “own destructive conflict behaviour” (M_Diff_ = 0.4), although the discrepancy in “partner support and recognition” is comparable. With regard to the parental role, the most substantial difference (M_Diff_ = 0.6) is evident for the item “unspecific strain”. The two groups of mothers differ most in their satisfaction with various aspects of life, with mean differences ranging from 0.9 to 1.0.

**Table 2:** Results of the t-tests for independent samples, mentally stressed and mentally not stressed mothers

***n***	**Mental Health (MCS SF-12)**				
	impaired (MCS <40)		not impaired (MCS ≥40)		*p*
	153		1,288		
	M	SD	M	SD	
Partnership					
Disagreements with partner	2.5	.785	2.1	.692	<.001
Own destructive conflict behaviour	2.5	. 797	2.1	.717	<.001
Partner support and recognition	4.1	. 939	4.4	.706	<.001
Parental role					
Parental competence	3.8	.571	4.1	.534	<.001
Unspecific strain	3,0	1,004	2.4	.943	<.001
Missing autonomy	2.5	1.031	1.9	.852	<.001
Overprotectiveness	2.8	.947	2.4	.884	<.001
Satisfaction with					
work-life balance	5.6	2.463	6.5	2.069	<.001
	*n*=111		*n*=940		
leisure activities, hobbies, interests	5.7	2.146	6.7	2.041	<.001
friends, acquaintances, social contacts	6.6	2.226	7.5	1.940	<.001
family	8.0	1.864	8.8	1.378	<.001

### Linear regression

Table 3 shows the results of the linear regression with 1.441 mothers, controlling for age, number of all children in the household, age of youngest cohabiting child, education, weekly working hours, migration background and net equivalence income (according to OECD). The overall model is significant, so that the examined variables explain the mother’s psychological distress as measured by the MCS (F(21, 1249) = 19,37, *p* <.001). The R^2^ for the overall model is 0.22 (adjusted R^2^ = 0.21), indicating medium to high goodness of fit according to Cohen [39]. The findings of the study indicate that “parental competence” (B = 1.606, *p* <.001) and “satisfaction with family” (B = 0.432, *p* =.029) are positively associated with good mental health in the sense of increased MCS scores. Conversely, “disagreements with partner” (B=−1.318, *p* =.002), “own destructive conflict behaviour” (B=−1.232, *p* =.002), “unspecific strain” (B=−1.402, *p* <.001), “missing autonomy in the parenting role” (B=−0.732, *p* =.030), “overprotectiveness” (B=−1.015, *p* <.001), and “low satisfaction with work-life balance” (B=−2.537, *p* =.003) (in comparison to high) correlate with ill mental health, i.e. decreased MCS-Scores. Among the six control variables, two show minor associations with mothers’ mental health: Maternal stress decreases with growing maternal age (B = 0.108, *p* =.039) and decreasing age of the youngest child (B=−0.015, *p* =.020). Controls for smoking, alcohol use and physical activity showed significant associations with the mental health status of mothers’ in bivariate tests, but they did not in the additional regression model (not reported in table).

**Table 3:** Results from linear regression with MCS as dependent variable using bootstrapping (resampling of observation cases)

**Independent variables**	**Unstandardized B**	***p*** **-value**	95 % confidence interval of B* **(percentile)**	
(constant)	47.068	<.001	39.146	54.535
Partnership				
Disagreements with partner	-1.318	.002	-2.184	-.550
Own destructive conflict behaviour	-1.232	.002	-1.938	-.534
Partner support and recognition	-.156	0.687	-.914	.597
Parental role				
Parental competence	1.606	<.001	.724	2.564
Unspecific strain	-1.402	<.001	-1.983	-.854
Missing autonomy	-.732	.030	-1.419	-.111
Overprotectiveness	-1.015	<.001	-1.563	-.485
Satisfaction with				
work-life balance (reference: high)				
low	-2.537	.003	-4.140	-1.007
middle	-.431	.441	-1.516	.651
missing	-.418	.556	-1.990	.940
family	.432	.029	.034	.808
leisure activities. hobbies. interests	.177	.225	-.100	.471
friends. acquaintances. social contacts	.200	.216	-.115	.506
Covariates				
Age	.108	.039	.008	.212
Number of all children living with anchor	.546	.087	-.090	1.138
Age of youngest cohabiting child	-.015	.020	-.028	-.003
Education (reference: high)				
low	-.901	.338	-2.613	.878
middle	-.404	.367	-1.430	.573
Average weekly working hours	.048	.057	-.001	.096
Migration background (reference: no migration background)	-.158	0.751	-1.327	.924
Net equivalence income (according to OECD)	0	0.904	-.001	.001

B = Unstandardized Regression coefficient

*Values from bootstrapping (1,000 samples)

If the variables of the three areas partnership, parental role and satisfaction are entered separately into a regression model together with the controlling variables, the area parental role accounts for the most explanatory power ($\:{R}_{adj}^{2}$=0.160, *p* <.001), partnership for less ($\:{R}_{adj}^{2}$=0.104, *p* <.001) and satisfaction for the least ($\:{R}_{adj}^{2}$=0.083, *p* <.001).

## Discussion

This study identifies important aspects of women’s life regarding maternal mental health, i.e. their parental role, relationship with a partner, and satisfaction with various areas of life. Moreover, even if some of the differences are minor, the comparison of women with and without mental strain hints to mentally stressed mothers’ additional burdens.

The area parental role has in comparison to partnership and satisfaction the highest explanatory power for maternal mental stress. All four variables of the parental role show a significant correlation with maternal stress. Accordingly, parents who feel less safe in their parental role experience higher stress levels, whereas higher confidence is associated with less stress [40]. Furthermore, parental burdens express themselves in parents feeling overloaded, or trapped in the parental role, or torn between different roles [41]. The variable “unspecific strain” has the strongest power for maternal mental health, containing that the life with child(ren) is exhausting, and the situation may no longer be bearable. These two items represent an overwhelming exhaustion, which, at the same time, is a key symptom of parental burnout [42], a serious mental health problem among 1.5% of parent in Germany [43].

Regarding the role of partnership, “disagreements with the partner” and “own destructive conflict behavior” have shown significant associations with maternal mental health in the linear regression model. This finding is consistent with the literature, showing an association between partnership conflict and postnatal maternal depression [44, 45], and an association between paternal involvement, as well as the partner relationship and maternal mental health [46]. Furthermore, the present study indicates that partnership conflict is associated with maternal mental health outside the peri- and postnatal period. While the importance of partner support for maternal mental health has been documented elsewhere [14, 47], the impact of partner support and recognition is not significant in this model, suggesting that the absence of partner conflict or of destructive conflict behaviours may be more relevant than sole partner support for maternal mental health. This may be due to the inclusion of mothers with older children and the consideration of a longer period of motherhood. It may also be due to gender role expectations and stereotypes, which can change over time [48].

Further, the presented findings indicate that maternal satisfaction with family and work-life balance is associated with their mental health, a previously unreported relationship in the existent literature. Previous research has shown that low levels of family support and psychological distress in mothers are associated and that increased family support could be protective [49, 50]. In addition, poor work-life balance is associated with poor mental health, especially for women [51]. However, these studies do not include the relationship between mothers’ satisfaction with their work-life balance or with their family life and their mental health, thus this study adds an important perspective.

It has been demonstrated that maternal mental health is linked to conflicts in partnership, challenges in the parental role, and dissatisfaction with work-life balance and the family. While the findings are not exhaustive, they might give an orientation for how to understand and to support maternal mental health and for their inclusion in counselling and therapy. The same holds for lifestyle factors, as literature shows the importance of healthy lifestyle choices for individuals’ mental health [37]. Inpatient preventive or rehabilitation clinics might as well be able to use the insights of this study and further address aspects of the parental role, the relationship, and satisfaction with the examined aspects of life while treating their patients. Otto’s [52] research on the effects of inpatient preventive and rehabilitative measures for mothers and their children indicates that illnesses and psychological strains increase among mothers whose application for a mother-child measure has been accepted but who simultaneously have to wait more than half a year for admission. The efficacy of the measures has been demonstrated in a number of studies, with a concomitant improvement in maternal mental health being observed [25, 52–54]. In the course of the covid-19 pandemic, the measures have also attracted increased international attention [55].

Overall, the inextricable links between maternal mental health and satisfaction with various aspects of life, as well as roles as parents and partners, should provide valuable insights for health professionals and social workers.

### Strengths and limitations

By focusing on maternal mental health beyond the period of pregnancy, birth and postnatal phase this study expands an important field of research. Additionally, by exploring the context of maternal mental health in relation to specific topics, such as the parental role, partnership, and satisfaction with various aspects of life, it contributes to a more differentiated view on maternal mental health. The present study offers empirical evidence for associations between maternal mental health and family, partnership, and satisfaction, which so far have predominantly been discussed on a more theoretical basis in the literature. Finally, while the correlations of various independent variables on women’s mental health may appear negligible in a clinical context, they can be considered substantial in a psychosocial context, indicating potential starting points for prevention and intervention.

At the same time, the present study is subject to certain limitations. Firstly, previous research has shown that individuals with poorer health are often underrepresented in surveys [55, 56], which might also be suspected for mentally stressed mothers. However, this connection has not yet been demonstrated for pairfam participation. Secondly, in our attempt to highlight crucial factors associated with mothers’ mental health, we cannot claim comprehensiveness, as further major determinants of mental health could not be examined due to the absence of respective variables in the pairfam data set. Thirdly, even if the SF-12 is a well-established and validated instrument [30], its cut-off value is based on a norm sample ranging as far back as 1998 which does not offer a validated cut-off value particularly for women (or men). Additionally, the MCS does not comprehensively reflect the mental health status of respondents, and self-reports of the interviewed women may be influenced by their ambition to answer in a socially desirable manner. Furthermore, a panel effect based on the repeated interviews that preceded the 11th wave may have had an influence, and because the included variables change between the respective waves, there was no way to apply a larger number of previous waves. Still, it seemed adequate for the beginning to initiate the investigations by examining the general relevance of the variables in question through a cross-sectional analysis. Subsequent in-depth evaluations should be conducted using the more recent data releases in order to make use of the time series data and to draw conclusions regarding causalities. In summary, it would be preferable if the analyses were repeated with another measurement instrument for the mental health status of mothers, and with a longitudinal study design.

## Conclusion

This study underlines the significance of maternal mental health, highlighting its correlations with mothers’ experiences in the parental role, conflicts in partnerships, and satisfaction with work-life balance and family. Among these factors, the parental role has the strongest association with stress, with feelings of insecurity and overload leading to increased mental strain. Concurrently, the findings suggest that conflicts in partnership may exert a more substantial impact on maternal mental health than mere partner support. Furthermore, the study provides novel insights into the significance of satisfaction with work-life balance and family life, which have received less attention in previous research. The results emphasise the necessity for targeted preventive and rehabilitative measures for mothers experiencing psychological distress. In conclusion, this study demonstrates that a holistic approach is imperative, encompassing not only the parental role but also partnership dynamics and overall life satisfaction. It therefore seems reasonable to give these issues some more consideration, given that possible approaches have been confirmed as successful prevention and therapy to effectively support maternal mental health.

### Availability of data and materials

The data used is subject to a license and is not publicly available. The Dataset is available from GESIS (https://www.pairfam.de/en/data/data-access/) for scientific use.

## Supplementary Information

Additional file 1: Overview of the collection and transformation of the variables.pdf.

## Data Availability

Data is provided within the manuscript.
