# Correction: Exploring the connection between maternal mental health and partnership, parental role, and satisfaction with various aspects of life using pairfam data: a cross-sectional analysis

**DOI:** 10.1186/s12905-025-04106-2

**Published:** 2025-10-30

**Authors:** Monique Förster, Claudia Kirsch, Julia Habermann, Dorothee Noeres

**Affiliations:** Hannover Medical School, Medical Sociology Unit, Carl-Neuberg-Straße 1, Hannover, 30625 Germany

**Correction: Förster et al. BMC Women's Health 25, 395 (2025)**

**https://doi.org/10.1186/s12905-025-03933-7**

Following publication of the original Article [1], the following errors were reported. The original Article has been corrected.

**Table:** 

Section and Paragraph	Incorrect text	Correct text
Background	An error in the following sentence: "The most recent 12-month prevalence..." The year "201" and the percentage value "221%" are incorrect.	The corrected sentence should read: The most recent 12-month prevalence of mental disorders in Germany was 27.7%, in **2011**, with women demonstrating a higher prevalence compared to men (33.5% vs. **22.1**%) [4, 5].
Background	a typo error in the term "Matrnal" in the sentence beginning with "Matrnal mental health has…".	It appears that "Matrnal" should be "Maternal".
Background	In the sentence: "Furthermore, 16% f sickness absences were associated". It appears that the letter "o" is missing in the word "of".	The corrected sentence should read: "Furthermore, 16% **of** sickness absences were associated".
Background	A few incorrect terms in the following sentence: "Research indicates that up to 80% of **mother** in inpatient mother-child measures show clinically relevant psychological stress [24]. Consequently, 54.2% of the **mother** participating in such a measure demonstrate elevated stress levels, 26.2% exhibit heighened fear, and 23.0% encounter **augented** strain due to depression [25]."	The highlighted terms that need correction are: "mother" (should be "mothers"), and "augented" (should be "augmented"). The corrected sentence should read: “Research indicates that up to 80% of **mothers** in inpatient mother-child measures show clinically relevant psychological stress [24]. Consequently, 54.2% of the **mothers** participating in such a measure demonstrate elevated stress levels, 26.2% exhibit heighened fear, and 23.0% encounter **augmented** strain due to depression [25]."
Statistical analyses	In the sentence: “Testing the necessary assumptions…….” the letter "a" is missing in the word "and".	The corrected sentence should read: “Testing the necessary assumptions linearity, independent errors, homoscedasticity, normally distributed errors, multicollinearity [29], we found no assumptions violated, still we conducted bootstrapping (1,000 samples) with replacement for more robust con­fidence intervals (95%) **and** excluded user-missing values.
