# Supplementary material for: Exploring the connection between maternal mental health and partnership, parental role, and satisfaction with various aspects of life using pairfam data: a cross-sectional analysis
Source: BMC Womens Health. 2025 Aug 18;25:395. doi: 10.1186/s12905-025-03933-7 (PMC12363032; doi:10.1186/s12905-025-03933-7)
Supplement: Supplementary file 1 — Additional file 1: Overview of the collection and transformation of the variables.pdf. [file 12905_2025_3933_MOESM1_ESM.docx]

Additional file 1

Overview of the collection and transformation of the variables

Independent variables

| Construct | Item wording | Scale |
| --- | --- | --- |
| Partnership | | |
| Disagreements with partner | Mean score of:  How often do you and [name of current partner] disagree in the following areas?   - Spending leisure time - The division of chores between you and your partner (e.g., housework, child care) - Financial matters - Your involvement, or that of your partner, at work, training, school - The question of how you deal with each other (jealousy, fairness, consideration) - Parenting matters and questions of how to deal with children | 1 Almost never or never  up to  5 Very often |
| Own destructive conflict behavior | Mean score of:  What happens when you have a disagreement with [name of current partner]? Please indicate how often each of you engaged in the following behaviors. Please refer to the past six months.  How often did you engage in these behaviors?   - Insult or verbally abuse your partner - Remain silent - Yell at your partner - Refuse to talk about the subject - Feel instantly offended - Blame your partner, make him feel guilty | 1 Almost never or never  up to  5 Very often |
| Partner support and recognition | Mean score of:  How do you feel in your role as a parent? To what extent do the following statements apply to you?   - My partner supports me in caring for and parenting my child(ren). - I feel recognized in my role as a mother by my partner. | 1 Not at all  up to  5 Absolutely |
| Parental role | | |
| Parental competence | Mean score of:  How do you feel in your role as a parent? To what extent do the following statements  apply to you?   - I can meet the needs of my child(ren) very well. - I can put into practice what I resolve to do regarding child care and parenting. - It’s up to me how I manage child care and parenting. - I feel helpless concerning child care and parenting. | 1 Not at all  up to  5 Absolutely |
| Unspecific strain | Mean score of:  To what extent do the following statements apply to you and your child(ren)?   - My life with my child(ren) is exhausting. - I am often at the end of my rope. | 1 Not at all  up to  5 Absolutely |
| Missing autonomy | Mean score of:  To what extent do the following statements apply to you and your child(ren)?   - I have the feeling that taking care of my child(ren) takes up all my strength and that my whole life revolves around it. - When I am with my child(ren) there is nothing else I’d rather be doing. - I look forward to being with my child(ren). - I feel trapped by my parental duties. | 1 Not at all  up to  5 Absolutely |
| Overprotectiveness | Mean score of:  If you think of your child(ren), to what extent do the following statements apply to you?   - I am always worrying that something could happen to my child(ren). - I am always asking myself if I am doing the right thing for my child(ren). - Sometimes I cannot sleep at night because I imagine that something could happen to my child(ren). |  |
| Satisfaction with ... | | |
| … work-life balance | How satisfied are you with the proportion of time that you spend on the job or for your vocational training or university education relative to the time that you spend on your personal life? | 0 Very dissatisfied up to  10 Very satisfied |
| … leisure activities, hobbies, interests | How satisfied are you with the following domains in your life?   - Leisure activities, hobbies, interests | 0 Very dissatisfied up to  10 Very satisfied |
| … friends, acquaintances, social contacts | How satisfied are you with the following domains in your life?   - Friends, acquaintances, social contacts | 0 Very dissatisfied up to  10 Very satisfied |
| … family | How satisfied are you with the following domains in your life?   - Family | 0 Very dissatisfied up to  10 Very satisfied |

Dependent variable: SF-12 (Version 2.0)

|  | Question | Scale |
| --- | --- | --- |
|  | How would you describe your health status in the past 4 weeks? | 1 bad  2 Not so good  3 Satisfactory  4 Good  5 Very good |
|  | When you have to climb several flights of stairs on foot, does your health limit you greatly, somewhat, or not at all? | 1 Greatly  2 Somewhat  3 Not at all |
|  | And what about other demanding everyday activities, such as when you have to lift something heavy or do something requiring physical mobility: Does your health limit you greatly, somewhat, or not at all? | 1 Greatly  2 Somewhat  3 Not at all |
| When answering, please refer to the past four weeks. During this time, how often... | | |
|  | did you feel down and gloomy? | 1 Almost never  2 Sometimes  3 Often  4 Almost always |
|  | feel calm and composed? |  |
|  | feel full of energy? |  |
|  | have severe physical pain? |  |
|  | that due to physical health problems you achieved less than you wanted to at work or in everyday activities? |  |
|  | feel that due to physical health problems you were limited in some way at work or in everyday activities? |  |
|  | feel that due to mental health or emotional problems you achieved less than you wanted to at work or in everyday activities? |  |
|  | feel that due to mental health or emotional problems you carried out your work or everyday tasks less thoroughly than usual? |  |
|  | feel that due to physical or mental health problems you were limited socially, that is, in contact with friends, acquaintances, or relatives? |  |

8 subscales of the SF-12

- physical functioning (2 items)
- role physical (2 items)
- bodily pain (1 item)
- general health (1 item)
- vitality (1 item)
- social functioning (1 item)
- role emotional (2 items)
- mental health (2 items)

2 dimensions of the SF-12

- physical health composite scale (PCS)
- mental health composite scale (MCS)
